# What Is the Role of Therapeutic Plasma Exchange as an Adjunctive Treatment in Severe COVID-19: A Systematic Review

**DOI:** 10.3390/v13081484

**Published:** 2021-07-28

**Authors:** Łukasz J. Krzych, Zbigniew Putowski, Marcelina Czok, Mariusz Hofman

**Affiliations:** Department of Anaesthesiology and Intensive Care, Faculty of Medical Sciences in Katowice, Medical University of Silesia, 40-752 Katowice, Poland; putowski.zbigniew@gmail.com (Z.P.); mczok@poczta.fm (M.C.); mariuszhf@gmail.com (M.H.)

**Keywords:** SARS-CoV-2, COVID-19, therapeutic plasma exchange, cytokine storm, ARDS

## Abstract

Introduction: Since the COVID-19 pandemic outbreak, multiple promising treatment modalities have been tested, however, only several of them were proven to be effective. Therapeutic plasma exchange (TPE) has been recently discussed as a possible supportive treatment for severe cases. Methods: To investigate a possible role of TPE in severe COVID-19 we used a structured systematic search strategy to retrieve all relevant publications in the field. We screened in PubMed, EMBASE, Web of Science, Cochrane Library and clinicaltrials.gov for data published until the 4 June 2021. Results: We identified 18 papers, enrolling 384 patients, 220 of whom received TPE. The number of TPE sessions ranged from 1 to 9 and the type of replacement fluid varied markedly between studies (fresh frozen plasma or 5% albumin solution, or convalescent plasma). Biochemical improvement was observed in majority of studies as far as C-reactive protein (CRP), interleukin-6 (IL-6), ferritin, lactate dehydrogenase (LDH), D-dimer concentrations and lymphocyte count are concerned. The improvement at a laboratory level was associated with enhancement of respiratory function. Adverse effects were limited to five episodes of transient hypotension and one femoral artery puncture and thrombophlebitis. Conclusions: Although the effect of therapeutic plasma exchange on mortality remains unclarified, the procedure seems to improve various secondary end-points such as PaO_2_/FiO_2_ ratio or biomarkers of inflammation. Therapeutic plasma exchange appears to be a safe treatment modality in COVID-19 patients in terms of side effects.

## 1. Introduction

Since the COVID-19 pandemic outbreak, multiple promising treatment modalities have been tested, however, only several of them were proven to be effective. Mortality of critically ill COVID-19 patients remains high, depending on population characteristics [1,2,3].

One of frequently discussed pathomechanisms for the severe course of COVID-19 is an excessive immune response leading to proinflammatory cytokine storm (often similar to the course of macrophage activation syndrome) that is associated with multiorgan dysfunction [4]. Moreover, a heavily studied process is hypercoagulability induced by numerous mechanisms: SARS-CoV2 tropism towards ACE II receptors, excessive complement activation, production of harmful antibodies (often similar to those found in antiphospholipid syndrome), formation of immunological complexes, release of procoagulant factors (e.g., von Willebrand factor) and diffused endothelialitis [5,6,7,8,9]. Usually, lactate dehydrogenase (LDH), ferritin, interleukin-6 (IL-6), C-reactive protein (CRP) and D-dimers are discussed as the biomarkers for predicting the severity of the disease [10,11,12,13,14]. 

Therapeutic plasma exchange (TPE) is a procedure in which plasma is separated from the morphotic elements of blood and is then replaced by either albumin solution or fresh frozen plasma (FFP). The aim of TPE is to eliminate morbific factors, often pathological antibodies [15]. Myasthenia gravis, inflammatory demyelinating polyneuropathies, thrombotic microangiopathy or macrophage activation syndrome are only narrow examples of the applications of TPE [16]. In the latter diseases, the elimination of pathological antibodies reduces the procoagulable state and, therefore, improves survival of patients.

As organ injury is triggered by cytokine storm-mediated immune reaction, theoretically, the elimination of cytokines and harmful antibodies could attenuate the severity of the disease. Additional removal of fibrin degradation products (e.g., D-dimers) could also improve the hemostatic balance [17]. For those reasons, TPE has been recently discussed as a possible supportive treatment for severe COVID-19 cases [18]. The purpose of this review was to investigate efficacy and safety of TPE in severe COVID-19 in a systematic manner. Participants, interventions, controls and outcomes (PICO) criteria are presented in Table 1.

## 2. Methods

By following the PRISMA guidelines, we used a structured systematic search strategy to retrieve all relevant publications regarding TPE use in severe COVID-19 [19]. We screened for data that were published until 4 June 2021 in PubMed, EMBASE, Web of Science, Cochrane Library and clinicaltrials.gov. The search string was as follows: (plasmapheresis) OR (therapeutic plasma exchange) OR (total plasma exchange) OR (apheresis) OR (plasma exchange) AND (sars-cov-2) OR (coronavirus) OR (COVID-19). We excluded animal studies, papers not in the English language, non-original papers and case reports (but not case series). Duplicates were identified and excluded as well. The remaining records were screened by three independent investigators and full texts were retrieved if at least two adjudicators agreed to include the paper. Differences of opinion were resolved by a discussion. Then, available manuscripts were reviewed by all investigators and included into a comprehensive assessment if three adjudicators agreed that the study results were compliant with the goals of this review. If no agreement was reached, then a fourth reviewer made a final decision. For our analysis, we retrieved the following items from the included studies: authors, year of publication, type of a study, patient’s characteristics, concomitant therapies, time of TPE initiation and cessation, dose of TPE, type of replacement fluids, adverse effects associated with TPE and outcomes (change in inflammatory biomarkers concentrations, clinical changes and survival). We used the RoB2 tool for the assessment of the risk of bias of randomized controlled trials [20].

## 3. Results

### 3.1. Included Studies

**Figure 1:**
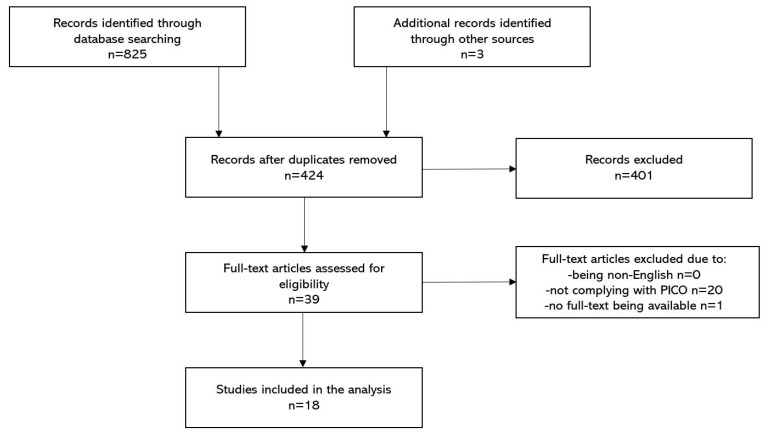
Structured search strategy.

By using the search string within various medical databases (presented in the Methods Section) we identified 825 articles in total. After removing duplicates (*n* = 401) we screened the remaining papers by evaluating titles and abstracts (*n* = 424). By using the PICO criteria and the inclusion and the exclusion criteria, we distinguished 39 papers for the full-text read assessment. After excluding some of the articles for numerous reasons, the final 18 papers were included in the systematic review. The most common types of studies were case-series studies (*n* = 14) [21,22,23,24,25,26,27,28,29,30,31,32,33,34], then case-control studies (*n* = 2) [17,35] and a propensity score matched study [36]. Only one randomized controlled trial was included in the analysis [37]. Study selection process is presented on the flowchart (Figure 1). A summary of published studies is shown in Table 2. Extended data regarding the included studies is presented in the Table S1.

### 3.2. Quality Assessment

Only one study was a randomized controlled trial [37]. The risk of bias of the study was rated as “low” by implementing the RoB2 tool (randomization process: low risk; deviations from the intended interventions: low risk; missing outcome data: low risk; measurement of the outcome: low risk; selection of the reported results: low risk). The remaining studies (*n* = 17) consisted mostly of case-series and observational data of limited populations. Therefore, we collectively defined the risk of bias of those studies as “high” due to methodological reasons.

### 3.3. Patient Characteristics

Out of 384 patients, 220 received TPE. The number of patients in the studies varied from 3 to 90 with a median of 8 patients (IQR 5–18) [17,21,22,23,24,25,26,27,28,29,30,31,32,33,34,35,36,37]. The mean or the median age of participants in seven studies was below 65 years [8,10,11,12,13,14,15], whereas in the remaining four studies, it was above 65 years [5,6,7,9]. Information regarding gender was available in all of the studies [17,21,22,23,24,25,26,27,28,29,30,31,32,33,34,35,36,37], of which the majority of patients were male. Only three studies had a similar gender ratio of about 40%–60% [22,30,33]. The most common reason to qualify a patient for TPE was acute respiratory distress syndrome (ARDS) (*n* = 8 [21,22,23,24,32,33,35,37]). Another frequent inclusion criterion was cytokine release syndrome (CRS) [23,25,36,37]. The mean or the median SOFA score on admission was provided in nine studies and varied between 5 and 12.3 [17,23,29,30,31,34,35,36,37]. Data regarding the frequency of invasive mechanical ventilation (and intubation) were available in 17 studies and varied between 16% and 100% [17,22,23,24,25,26,27,28,29,30,31,32,33,34,35,36,37]. 

### 3.4. Interventions

The information about days between the onset of COVID-19 symptoms and the initiation of TPE was provided in nine studies and varied from 6.5 to 39 days (median = 14 days) [21,22,23,24,26,29,32,33,37]. The number of TPE sessions varied between 1 and 9 [17,21,22,23,24,25,26,27,28,29,30,31,32,33,34,35,36,37]. The information regarding single dose of TPE was provided in 11 studies and the most common dosage varied between 1 and 1.5× of the patient’s plasma volume [21,22,23,24,26,28,30,31,33,36,37]. In terms of replacement fluid, FFP was used in nine studies [21,22,28,32,33,34,35,36,37], 5% albumin solution was used in five studies (in three of those studies, together with FPP) [23,25,27,29,30] and convalescent plasma was used in two studies [24,31]. One study did not provide information regarding the type of a replacement fluid [17]. In regard to other immunomodulatory treatments along with TPE, all studies but one implemented various pharmacological treatments [17,21,22,23,24,25,26,27,28,29,30,31,32,33,35,36,37]. The most common immunomodulatory drugs were corticosteroids [17,22,23,24,26,28,29,31,32,33,36,37].

### 3.5. Outcomes

The most frequent outcome reported in the studies was mortality. The studies varied in terms of the day of mortality assessment, however, the most frequent was day 28 (*n* = 5) [23,24,26,35,36], followed by day 14 (*n* = 3) [21,25,27]. The randomized controlled trial by Faqihi et al. reported mortality at day 35 [37]. In nine studies, no specified day of mortality assessment was provided [17,22,28,29,30,31,32,33,34]. The mortality varied across the studies, ranging from 0% to 60% (median = 18.35%, IQR: 8.3%–28.6%). Four studies reported differences in mortality between patients who received TPE and those who did not receive it (8.3% vs. 58.3%, 0% vs. 35%, 8.9% vs. 38.5% and 20.9% vs. 34.1%) [17,35,36,37]. The median concentrations of pre-TPE and post-TPE biomarkers provided by the studies are presented in Table 3.

In a randomized controlled trial by Faqihi et al., the overall mortality difference was not significant (20.9% vs. 34.1%; *p* = 0.09); however, the median length of stay and duration of mechanical ventilation reached statistical significance. Additionally, PaO_2_/FiO_2_, Lymphocyte count, IL-6, LDH, D-Dimers, Ferritin and ADAMTS-13 activity changed significantly after the implementation of TPE (Table S1) [37].

### 3.6. Side Effects

Data regarding the side effects of TPE were available in 10 studies [23,24,25,26,28,29,34,35,36,37]. Most of those studies reported no side effects related to the use of TPE [23,25,28,29,34,37]. The most frequent side effect was hypotension (which occurred in five patients across all the populations from the studies) [24,26,35]. There was one episode of femoral artery puncture and thrombophlebitis [36]. There were no deaths associated with the procedure.

## 4. Discussion

This systematic review focused on summarizing the data concerning the role of TPE in severe COVID-19 infection (as of June 2021). Based on the papers included in this review, we cannot produce a clear message regarding the effect that TPE has on mortality. However, the surrogate endpoints such as improvements in various biomarkers (CRP, LDH, D-Dimers, Ferritin, IL-6, PaO_2_/FiO_2_ ratio, etc.) seem to be well documented and are consistent among the studies. The frequency of side effects related to TPE is low. The considerable heterogeneity within and among the studies, and the fact that the majority of them were case-series studies, limits drawing definite conclusions. 

A considerable number of included studies presented a positive effect of TPE on mortality. However, the majority of those studies were case-series or case-control studies of limited populations. Such a strong effect in that many studies (even a 0% mortality rate in critically ill patients) most probably is a result of a publication bias. Indeed, the only RCT (of low risk of bias) included in this review failed to deliver significant results regarding mortality (the study might have been underpowered as it was terminated due to low patient recruitment). However, the study was able to present a significant reduction in days of mechanical ventilation or hospital length of stay. 

It must be pointed out that the vast majority of patients who underwent TPE among the studies were intubated and often suffered from septic shock and progressing multi-organ failure. As intubation in COVID-19 patients is often related with the compromised prognosis, we speculate that it is possible that the implementation of TPE in such a critical condition is no longer able to restore homeostasis [38]. For example, in the PLEXIT study, TPE was initiated mostly in non-invasively ventilated patients. In that study, the patients underwent the TPE procedure on the basis of CRS recognition (higher levels of ferritin, CRP, D-dimers, LDH and lymphopenia) [36]. Therefore, we may assume that signs and symptoms of rapid deterioration in organ function, including respiratory failure, could serve as indications for TPE application. The SOFA score may be applied as an easy-to-use method of multi-organ failure assessment. Such a hypothesis could be tested in the future, well-designed studies.

In regards to biochemical improvements, the summary of those is presented in Table 3. Despite the values not being weighted, they provide a rather clear trend of changes that occurred within various biomarkers. As LDH, ferritin, IL-6, CRP and D-dimers are discussed as the biomarkers in predicting severity of the disease, one may speculate that perhaps those molecules could serve as indicators for TPE initiation [10,11,12,13,14]. However, the trigger points would still be unknown. Based on previous data, the patients may benefit the most from removing IL-6. The molecule works as a procoagulant cytokine and probably is one of the factors that accounts for microvascular thrombosis in the course of infection [39]. Interestingly, Guiaro et al. published a study in which they provided a cut-off point of 35 pg/mL of IL-6 that was associated with an increased risk of mortality and ICU admission [40]. Perhaps such a cut-off point could be discussed as one of the possible trigger points for TPE. In our review, the median value of IL-6 concentration prior to TPE initiation obtained from the included studies was 118.7 pg/mL (IQR 25.2–295.3). 

Perhaps LDH removal is of particular importance as well. Its concentrations correlate with the release of proinflammatory molecules and lymphocyte count, which corresponds with the severity of the disease [41]. Increase of LDH may be harmful by production of lactate and enhancement of immune-suppressive cells and inhibition of natural killer (NK) cells and cytotoxic T-lymphocytes. The similar issue regards ferritin. Its elevated levels were found in patients with macrophage activation syndrome (MAS) and cytokine storm. Increase in ferritin concentration negatively impacts immunological condition as it plays a role in the inflammation process through its binding with the T-cell immunoglobulin and the expression of multiple proinflammatory mediators [42]. Noteworthily, severe COVID-19 patients may produce procoagulant antibodies, such as lupus anticoagulants and antibodies found in antiphospholipid syndrome [43]. 

TPE safety has been confirmed in the past [44,45,46]. In our analysis, adverse effects during TPE in COVID-19 were rather anecdotal, but we still need to bear in mind the limited number of observations and a considerable number of papers in which no information regarding side effects was provided. Importantly, none of the presented side effects posed a threat to a patient that could not be properly handled. The most common adverse effect in patients undergoing TPE are urticaria, hypocalcemia, rigors and headaches. More concerns were related to complications of the central venous catheter insertion site. None of them appeared in the included papers. 

## 5. Limitations

Firstly, a limited number of trials have been completed so far. Due to methodological heterogeneity between publications and subsequent risk of bias, we failed to prepare a meta-analysis. Case studies usually describe positive data; therefore, strong publication bias exists and the above-mentioned studies should be interpreted with caution. Only one randomized controlled trial has been performed [37]. The included studies suffer from lack of adequate reporting, namely, day of mortality assessment, side effects or median time from symptoms to TPE initiation. Furthermore, the procedure in COVID-19 has not been standardized yet. Clinical indications, time of commencement of TPE, number of sessions, time gaps between them and the type of replacement fluid varied between studies. Noteworthily, the type of a replacement fluid is debatable. Albumin solutions may cause significant and unpredictable disturbances of blood coagulation due to loss of pro- as well as anticoagulant factors [47,48]. One may then expect that convalescent plasma should be the first choice of treatment [49,50], but recent data are not so convincing [51]. Moreover, plasma transfusion may have serious side effects, including transfusion-related immunomodulation and transfusion-related lung injury [52], which are of particular importance in COVID-19 patients with respiratory failure. Moreover, the time at which various biomarkers’ concentrations were measured was different between the studies. So, reliable assessment of TPE efficacy requires the unification of procedure-related issues. In the future, this should be clarified. Lastly, TPE is never used as the sole treatment option. It is only a part of the complex patient-oriented multifactorial therapy.

## 6. Conclusions

Although the effect of therapeutic plasma exchange on mortality remains unclarified, the procedure seems to improve various secondary end-points such as PaO_2_/FiO_2_ ratio or biomarkers of inflammation. Therapeutic plasma exchange appears to be a safe treatment modality in COVID-19 patients in terms of side effects.

## Figures and Tables

**Table 1:** The PICO criteria used in the study.

**Participants**	Patients with severe course of COVID-19.
**Interventions**	Therapeutic plasma exchange (any type) as an adjunctive treatment.
**Control**	Due to limited number of studies and their methodological type, a control group was not required to include in the study.
**Outcomes**	Mortality and changes in various biomarkers, along with which additional attention was given to safety issues in retrieved papers.

**Table 2:** Summary of the included studies.

Author	Study Type	Population	Intervention	Median Time from First Symptoms to TPE Initiation	Replacement Fluid	Adverse Effects of TPE	Outcome
Zhang et al. [21]	Case-series	3 severely ill patients	1 TPE session	15 days	FFP	N/A	Mortality (day 14): 0%
Morath et al. [22]	Case-series	5 patients with COVID-19-induced multi-organ failure and ARDS	All patients received 1–2 TPE sessions	12 days	FFP	N/A	Mortality: 20%
Faqihi et al. [23]	Case-series	10 patients with ARDS, APACHE II score >20, septic shock or cytokine release syndrome	All patients received 5–7 TPE	6.5 days	5% albumin or FFP	None	Mortality (day 28): 10%
Gucyemetz et al. [17]	Case-control	73 patients with COVID-19-related pneumonia	18 patients received 3 TPE sessions	N/A	N/A	N/A	Mortality (non-TPE vs. TPE): 58.3% vs. 8.3% *
Khamis et al. [35]	Case-control	31 critically ill patients with COVID-19-related ARDS, severe pneumonia, septic shock or multiple organ dysfunction syndrome	11 patients underwent 5 TPE sessions	N/A	FFP	One hypotension episode treated with fluid bolus and hydrocortisone	Mortality (non-TPE vs. TPE, day 28): 35% vs. 0% *
Jaiswal et al. [24]	Case-series	14 patients with severe COVID-19 infection according to WHO classification	All patients received 1 TPE session	9 days	Convalescent Plasma	3 cases of hypotension treated with fluid bolus	Mortality (day 28): 28.6%
Gluck et al. [25]	Case-series	10 patients with COVID-19 and Penn class 3 and 4 cytokine release syndrome	All patients received 5 TPE sessions	N/A	5% albumin or FFP	None	Mortality (day 14): 0%
Karman et al. [36]	PSM	90 patients with severe COVID-19 infection and cytokine release syndrome	45 patients received one TPE until resolution of the disease	N/A	FFP and normal saline in 2:1 ratio	1 femoral artery puncture and thrombophlebitis treated accordingly	Mortality (non-TPE vs. TPE, day 28): 38.5% vs. 8.9% *
Fernandez et al. [26]	Case-series	4 critically ill patients with COVID-19	2–6 plasma exchange sessions	20 days	5% albumin + FFP	1 episode of hypotension and tachycardia	Mortality (day 28): 0%
Dogan et al. [27]	Case-series	6 patients with COVID-19–related autoimmune meningoencephalitis	1–9 plasma exchange sessions	N/A	5% albumin	N/A	Mortality (day 14): 16.7%
Adeli et al. [28]	Case-series	8 patients	3–5 plasma exchange sessions	N/A	FFP + albumin solution + calcium gluconate	None	Mortality (no specified day): 12.5%
De Prost et al. [29]	Case-series	4 critically-ill patients with high blood concentrations of neutralizing autoantibodies against type I interferons	3–4 plasma exchange sessions	18 days	5% albumin solution	None	Mortality (no specified day): 50%
Faqihi et al. [37]	RCT	87 intubated patients with either ARDS, APACHE II score >20 pts, septic shock or cytokine release syndrome	43 patients received 1–5 (median 3) plasma exchange sessions	8 days	FFP	None	Mortality (TPE vs non-TPE, day 35): 20.9% vs. 34.1 % (*p* = 0.09)
Hashemian et al. [30]	Case-series	15 patients	1–3 TPE sessions	N/A	5% albumin solution + 0.9% NaCl/convalescent plasma	N/A	Mortality (no specified day): 40%
Keith et al. [31]	Case-series	8 patients	1–7 plasma exchange sessions	N/A	FFP	N/A	Mortality (no specified day): 25%
Matsushita et al. [32]	Case-series	5 patients with PaO_2_/FiO_2_ ratio of less than 200 mmHg and/or labored respiration and/or tracheal intubation	3–7 plasma exchange sessions	14 days	FFP	N/A	Mortality (no specified day): 60%
Roshandel et al. [33]	Case-series	5 COVID-19 patients with respiratory failure	2 standard plasma exchange sessions	39 days	FFP + 5% albumin, then 0.9% NaCl/convalescent plasma	N/A	Mortality (no specified day): 20%
Truong et al. [34]	Case-series	6 critically ill patients with plasma hyperviscosity	2–3 plasma exchange sessions	N/A	FFP	None	Mortality (no specified day): 50%

Results presented in the “outcome” column are median. * results that were statistically significant. Absence of “*” means that the result was either not significant or the significance was not calculated; PaO_2_: partial pressure of oxygen; FiO_2_: fraction of inspired oxygen; ARDS: Acute Respiratory Distress Syndrome; SOFA—Sequential Organ Failure Assessment; APACHE II—Acute Physiology and Chronic Health Evaluation II; TPE—therapeutic plasma exchange; RCT–randomized controlled trial; PSM: propensity score matching; N/A: information not available.

**Table 3:** Median and interquartile ranges of values of various biomarkers measured before and after TPE.

Parameter	Values: Median (IQR)
pre-TPE PaO_2_/FiO_2_ (mmHg)	132 (112.5–153.5) [21,23,24,25,29,30,35,37]
post-TPE PaO_2_/FiO_2_ (mmHg)	224 (216.5–300) [21,23,24,29,30,35,37]
pre-TPE CRP (mg/L)	132 (79–168.5) [17,21,22,23,24,25,26,27,29,30,31,33,34,35,36,37]
post-TPE CRP (mg/L)	28.5 (11.1–47.5) [17,21,22,23,24,25,30,31,33,34,35,37]
pre-TPE Lymphocytes (10^9^/L)	0.7 (0.58–1.0) [17,21,23,24,25,26,29,35,37]
post-TPE Lymphocytes (10^9^/L)	1.04 (1.0–1.5) [17,21,23,24,35,37]
pre-TPE IL-6 (pg/mL)	118.7 (25.2–295.3) [17,21,22,23,25,26,27,32,35,36,37]
post-TPE IL-6 (pg/mL)	18.5 (5.7–35) [17,21,22,23,26,30,33,35]
pre-TPE LDH (U/L)	576.5 (492.5–849.5) [17,21,22,23,26,27,33,36,37]
post-TPE LDH (U/L)	245.5 (236–440) [17,22,23,26,33,37]
pre-TPE D-Dimers (mg/L)	6.05 (4.5–7.6) [17,22,23,24,25,26,27,28,29,31,34,35,36,37]
post-TPE D-Dimers (mg/L)	2.6 (1.3–4.0) [17,22,23,24,26,31,33,34,35,37]
pre-TPE Ferritin (ug/L)	1332 (1125–1444) [17,22,23,24,26,27,30,31,35,36,37]
post-TPE Ferritin (ug/L)	494 (352–842) [17,22,23,24,26,30,31,35,37]

The values presented on the table are not weighted. The values included in the table are provided in the Table S1.

## Data Availability

Not applicable.

## Supplementary Materials

The following are available online at https://www.mdpi.com/article/10.3390/v13081484/s1, Table S1: Detailed summary of the included studies.
