# Supplementary material for: What Is the Role of Therapeutic Plasma Exchange as an Adjunctive Treatment in Severe COVID-19: A Systematic Review
Source: Viruses. 2021 Jul 28;13(8):1484. doi: 10.3390/v13081484 (PMC8402853; doi:10.3390/v13081484)
Supplement: Supplementary file 1 [file viruses-13-01484-s001.zip › viruses-1304171-supplementary.pdf]

Table S1. Detailed summary of the included studies.

	Study Type	Population	Concomitant immunodulatory therapies	Intervention	Median time from first symptoms to TPE initiation	Replace ment fluid	Adverse effects of TPE	Outcome
Zhang et al. [21]	Case series	3 severely ill patients (median 55 year, all male) with COVID-19 related ARDS unresponsive to initial antiviral treatment and other therapeutic interventions.	Arbidol Interferon alpha-2b	1 TPE session (3L of plasma exchanged each session) 1-3 days after admission to ICU	15 days	FFP	N/A	**Mortality (day 14)** – 0%<br><br>**Values before and after TPE:**<br><br>**PaO_2/FiO_2** - 168 => 300 *<br>**CRP** – 123.1 => 19.4 mg/L *<br>**Lymphocytes** – 0.56 => 1.53 10^9/L<br>**IL-6** – 18.28 => 4.53 pg/mL<br>**LDH** – 823 => 303 U/L
Morath et al. [22]	Case series	5 patients (median 67 years, 60% male) with COVID-19 induced multi-organ failure and ARDS. All patients underwent IMV.	Hydroxychloroquine<br>Maraviroc<br>Aciclovir<br>Ganciclovir<br>Tocilizumab<br>Prednisolone<br>Convalescent serum	All patients received 1-2 TPE sessions (with median 3.39 L of plasma replaced each session)	12 days	FFP	N/A	**Mortality** – 20%<br><br>**Values 2 hrs before and 24 hrs after the last TPE:**<br><br>**CRP** – 126 => 50 mg/L*<br>**IL-6** – 410 => 102 pg/mL*<br>**LDH** – 651 => 440 U/L *<br>**D-Dimer** – 15 => 4 mg/L*<br>**Ferritin** – 1444 => 700 ug/L*
Faqihi et al. [23]	Case series	10 patients (median 51 years, 70% males) with either ARDS, APACHE II score >20, septic shock or cytokine release syndrome. On admission, the median SOFA score was 11 points. All patients underwent IMV.	Hydrocortisone<br>Hydroxychloroquine	All patients received 5-7 TPE (dosed as 1-1.5 plasma volumes each session)	6.5 days	5% albumin or FFP if patient presented coagulopathy	None	**Mortality (day 28)** – 10%<br><br>**Values 24 hrs before and 24 hrs after the last TPE:**<br><br>**PaO_2/FiO_2** - 110 => 340 mmHg *<br>**CRP** – 71.3 => 13.2 mg/L*<br>**Lymphocytes** – 0.6 => 1.15 10^9/L*<br>**IL-6** – 159.5 => 31.2 pg/mL*<br>**LDH** – 576.5 => 199.5 U/L*<br>**D-Dimer** – 7.4 => 0.9 mg/L*<br>**Ferritin** – 1233 => 290 ug/L*

Gucyemetz et al. [17]	Case-control	73 patients (aged ~ 62 years, 70% male) with COVID-19 related pneumonia. On admission, the median SOFA score was 6 points. 80% of patients underwent IMV.	Cytokine Filters in 7 patients<br>IL-6 blocker in 21 patients<br>Steroids in 41 patients	18 patients received 3 TPE sessions	N/A	N/A	N/A	**Mortality (non-TPE vs TPE)** – 58.3% vs 8.3%*<br><br>**Values before and after TPE:**<br><br>**CRP –** 118 => 9 mg/L<br>**Lymphocytes** – 0.9 => 1.02 10^9/L<br>**IL-6** – 161 => 24.5 pg/mL<br>**LDH** – 436 => 239 U/L<br>**D-Dimer** – 7.8 => 1.3 mg/L<br>**Ferritin** – 1268 => 405 ug/L
Khamis et al. [24]	Case-control	31 critically ill patients (mean age of 51 years, 90% male) with COVID-19 related ARDS, severe pneumonia, septic shock or multiple organ dysfunction syndrome. The median SOFA score on admission was 5 points. Out of 11 patients, who received TPE, only one was not intubated.	Tocilizumab in 12 patients	11 patients underwent 5 TPE sessions	N/A	FFP	One hypotension episode treated with fluid bolus and hydrocortisone	**Mortality (non-TPE vs TPE, day 28)** – 35% vs 0%*<br><br>**Values at day 0 and day 7:**<br><br>**SOFA score** – 6 => 4<br>**PaO_2/FiO_2** - 130.5 => 216.5 mmHg<br>**CRP** – 187 => 33 mg/L<br>**Lymphocytes** – 0.81 => 1.5 10^9/L<br>**IL-6** – 180.5 => 57 pg/Ml<br>**D-Dimer** – 3.4 => 2 mg/L<br>**Ferritin** – 1125 => 429 ug/L
Jaiswal et al. [25]	Case-control	14 patients (of median age 51.5 years, male 78.6%) with severe COVID-19 infection according to WHO classification (ARDS, sepsis or septic shock). All patients underwent IMV.	Methylprednisolone in all patients<br>Hydroxychloroquine in 5 patients<br>Favapiravir in 6 patients<br>Lopinavir/ritonavir in 10 patients	All patients received 1 TPE session (30-40ml/kg body weight of plasma exchanged each session)	9 days	Convalescent Plasma	3 cases of hypotension treated with fluid bolus	**Mortality (day 28)** – 28.6%<br><br>**Values at day 0 and day 7:**<br><br>**PaO_2/FiO_2** - 138.9 => 224.8 mmHg<br>**CRP** – 86.7 => 30.6 mg/L*<br>**Lymphocytes** – 0.7 => 1.04 10^9/L<br>**D-Dimer** – 4.20 => 4.21 mg/L<br>**Ferritin** – 1416.3 => 1051.4 ug/L
Gluck et al. [26]	Case-series	10 patients (mean age of 51.8 years, male 30%) with COVID-19 and Penn class 3 and 4 cytokine release syndrome.	Hydroxychloroquine in 2 patients	All patients received 5 TPE sessions	N/A	5% albumin or FFP if coagulopathy was present	None	**Mortality (day 14)** - 0%<br><br>**Values at day 0 and day 14:**

		60% of patients underwent IMV.						**PaO_2/FiO_2** - 115 => 151 mmHg<br>**CRP** – 149.9 => 24.8 mg/L*<br>**IL-6** – 32.04 => 5.92 pg/mL*
Karman et al. [27]	Propensity score matched study	90 patients (median age 70 years, male 92%) with severe COVID-19 infection and cytokine release syndrome (patients with septic shock at the time of admission and patients who died within 48 hours since admission were excluded from the analysis). 59.2% of patients were defined as critical (SOFA >10 points or evidence of septic shock). 16% of patients received IMV.	Methylprednisolone in all patients	45 patients received one TPE until resolution of the disease (1.5x of plasma volume exchanged each session)	N/A	FFP and normal saline in 2:1 ratio	1 femoral artery puncture and thrombophlebitis treated accordingly	**Mortality (non-TPE vs TPE, day 28)** – 38.5% vs 8.9%*<br><br>**Values at day 0:**<br><br>**CRP** – 145 => N/A mg/L<br>**Lymphocytes** – 0.7 => N/A 10^9/L<br>**IL-6** – 78 => N/A pg/mL<br>**LDH** – 549 => N/A U/L<br>**D-Dimer** – 3.5 => N/A mg/L<br>**Ferritin** – 1500 => N/A ug/L
Fernandez et. al. [28]	Case-series	4 critically ill patients (median age of 56 years, male 100%) with COVID-19. 75% of patients underwent IMV.	Lopinavir/ritonavir<br>Hydroxychloroquine<br>Interferon beta-1a<br>Tocilizumab<br>Dexamethasone<br>Methylprednisolone	2-6 plasma exchange sessions (1.2x plasma volume exchanged each session)	20 days	5% albumin + FFP	1 episode of hypotension and tachycardia requiring increase in norepinephrine doses following the FFP infusion	**Mortality (day 28)** - 0%<br><br>**Values at day 0 and day 14:**<br><br>**CRP** – 51.5 => 5.4 mg/L<br>**Lymphocytes** – 1.25 => 0.95 10^9/L<br>**IL-6** – 17.5 => 12.5 pg/mL<br>**LDH** – 551 => 245.5 U/L<br>**D-Dimer** – 11.76 => 3.65 mg/L<br>**Ferritin** – 2058 => 494 ug/L
Dogan et al. [29]	Case-series	6 patients (median age of 53 years, male 83%) with COVID-19–related autoimmune meningoencephalitis. All patients underwent IMV.	Lopinavir/ritonavir<br>Hydroxychloroquine	1-9 plasma exchange sessions	N/A	5% albumin	N/A	**Mortality (day 14)** – 16.7%<br><br>**Values at day 0:**<br><br>**CRP** – 138.6 => N/A mg/L<br>**IL-6** – 510 => N/A pg/mL

								**LDH** – 359 => N/A U/L<br>**D-Dimer** – 6.4 => N/A mg/L<br>**Ferritin** – 1332 => N/A ug/L
Adeli et al. [30]	Case series	8 patients (male 87.5%) of median age of 52 years. 37.5% of patients received IMV.	Dexamethasone, Hydroxychloroquine, Lopinavir/ritonavir, Interferon beta	3-5 plasma exchange sessions (2L of plasma exchanged each session)	N/A	A combination of FFP, Albumin solution and calcium gluconate	None	**Mortality (no specified day)** – 12.5%
De Prost et al. [31]	Case series	4 critically-ill patients (median age 52 years; 75% male) with high blood concentrations of neutralizing autoantibodies against type I interferons. Median duration of symptoms was 5.5 days. Median admission SOFA score was 7 points. 75% of patients underwent IMV.	Dexamethasone	3-4 plasma exchange sessions	18 days	5% albumin solution	None	**Mortality (no specified day)** – 50%<br><br>**Values at day 0:**<br><br>**PaO_2/FiO_2** - 70 => N/A mmHg<br>**Lymphocytes** – 1.1 => N/A<br>**D-Dimer** – 3.8 => N/A mg/L
Faqihi et al. [32]	Randomized controlled trial	87 intubated patients with either ARDS, APACHE II score >20, septic shock or cytokine release syndrome. The median age was 49 years. 82 % of all patients were male. The median SOFA score on admission was 10. Despite randomization, intervention group (43 patients) presented significantly higher frequency of pulmonary embolism and higher risk of developing	Dexamethasone, Ribavirin	43 patients received 1-5 (median 3) plasma exchange sessions (with doses of 1.5x plasma volumes on the first day and 1x plasma volume during the next days)	8 days	FFP	None	**Intervention group vs Control group:**<br><br>**Mortality (on day 35)** – 20.9% vs 34.1 % (p=0.09)<br><br>**ICU LOS** – 19 days vs 26 days *<br><br>**MV duration** – 15 days vs 19 days *<br><br>**SOFA score** – 10 => 2 points vs 9 => 4.5 points *<br><br>**Values at day 0 and within 24 hrs after finishing the last TPE:**<br><br>**PaO_2/FiO_2** - 135 => 300 mmHg vs 125 => 255 mmHg *

		cytokine release syndrome						**CRP** – 246 => 45 mg/L vs 234 => 78 mg/L * <br>**Lymphocytes** – 0.5 => 1.0 10^9/L vs 0.6 -=> 0.7 10^9/L * <br>**IL-6** – 458 => 35 pg/mL vs 122 => 27 pg/mL * <br>**LDH** – 876 => 236 U/L vs 378 => 343 U/L * <br>**D-Dimers** – 4.9 => 0.9 mg/L vs 2.5 => 0.95 mg/L * <br>**Ferritin** – 987 => 299 ng/mL vs 320 => 287 ng/mL * <br>**ADAMTS-13 activity** – 17 => 42% vs 37 => 32% *
Hashemian et al. [33]	Case-series	15 patients of mean age 57.6 years. (60% males) At baseline the mean SOFA score was 9.6. 27% of patients (with PaO2 /FiO2 lower than 100 mmHg) required IMV whilst NIPPV was used for the other subjects (73%). During the study, two patients on NIPPV developed severe hypoxemia requiring intubation.	Favipiravir, Remdesivir	1-3 TPE sessions (40 ml/ kg body weight of plasma exchanged each session)	N/A	5% albumin solution + 0.9% saline / convalescent plasma	N/A	**Mortality (no specified day)** – 40% <br>**Values before and after the last TPE:** <br>**PaO_2/FiO_2** 184.3 => 224.0 mmHg * <br>**CRP** – 47.3 => 28.5 mg/L * <br>**IL-6** – 8.3 => 5.7 pg/mL * <br>**Ferritin** – 1027.3 => 654.0 ng/mL*
Keith et al. [34]	Case series	8 patients (median age of 67.5 years; 75% male) with mean admission SOFA 6.8 points. 7 patients received IMV and 1 NIPPV.	Methylprednisolone, Hydroxychloroquine, azithromycin, zinc, tocilizumab	1-7 plasma exchange sessions (1x plasma volume during each session)	N/A	FFP	N/A	**Mortality (no specified day**) – 25% <br>**Values before and after the earliest TPE:** <br>**SOFA score** – 9.3 => 6.4 points * <br>**CRP** – 266.1 => 176.5 mg/dL *

								**D-Dimers** – 6.2 => 3.2 mg/L<br><br>**Ferritin** – 1404.9 => 984.4 ng/mL *
Matsushita et al. [35]	Case-series	5 patients (median age 76 years; 80% males,) with PaO2/FiO2 ratio of less than 200 and/or labored respiration and/or tracheal intubation (60% of patients underwent IMV).	Different combinations of methylprednisolone / remdesivir/ Favipiravir/ Cyclesonid /Gamma-globulin/ Nafamostat mesylate/ Hydroxychloroquine	3-7 plasma exchange sessions	14 days	FFP	N/A	**Mortality (no specified day)** – 60%
Roshandel et al. [36]	Case series	5 COVID-19 patients (median age 62 years; 40% male) with respiratory failure (respiratory rate >30/min, need for MV, oxygen saturation <90%). 20% of all patients underwent IMV.	Lopinavir/ritonavir, hydroxychloroquine, azithromycin and steroids	2 standard plasma exchange sessions (1.5-2.0 L exchanged each session) and 1 subsequent TPE with convalescent plasma	39 days	FFP + 0.9 % saline solution containing 5 % human albumin during the first 2 procedures, then convalescent plasma was used instead of FFP	N/A	**Mortality (no specified day)** – 20%<br><br>**Values before and after 7 days after TPE + CPT:**<br><br>**CRP** – 20 => 5 mg/L<br><br>**IL-6** – 55.16 => 3.66 pg/mL<br><br>**LDH** – 1124 => 576 U/L<br><br>**D-Dimer** – 3.8 => 1.6 mg/L
Truong et al. [37]	Case series	6 critically ill patients (of median age 54 years; 100% male) with plasma hyperviscosity. Mean SOFA score on admission was 12.3 points. 100% of patients underwent IMV.	N/A	2-3 plasma exchange sessions	N/A	FFP	None	**Mortality (no specified day)** – 50%<br><br>**Values 24 hrs before and 24 hrs after the last TPE:**<br><br>**D-dimers** - 5.9 => 4.8 ng/mL<br><br>**CRP** - 292 => 84 mg/L

*Results presented in the "outcome" column are median. * - results that were statistically significant. Absence of "*" means that the result was either not significant or the significance was not calculated. MV - mechanical ventilation; IMV - invasive mechanical ventilation; NIPPV – non-invasive positive pressure ventilation; SOFA – Sequential Organ Failure Assessment; APACHE II - Acute Physiology and Chronic Health Evaluation II; TPE – therapeutic plasma exchange; CPT - convalescent plasma transfusion; CRP – C-reactive protein, LDH – lactate dehydrogenase, IL-6 – interleukin 6, PaO_2 – partial pressure of arterial oxygen, FiO_2 – fraction of inspired oxygen, N/A – not available.*
